# The role of social influence on COVID-19 vaccination hesitancy and acceptance in Tanzania

**DOI:** 10.4102/jphia.v16i3.704

**Published:** 2025-04-18

**Authors:** Magolanga Shagembe, Chima E. Onuekwe, Egidius Kamanyi, Ambrose T. Kessy, Tumaini Haonga, William M. Mwengee

**Affiliations:** 1 Department of Sociology and Anthropology, College of Social Sciences, University of Dar es Salaam, Dar es Salaam, United Republic of Tanzania; 2 Department of Immunizations, Emergency Preparedness and Response (EPR), World Health Organization, Dar es Salaam, United Republic of Tanzania; 3 Centre for Health and Allied Legal and Demographical Development, Research and Training (CHALADDRAT), Nnamdi Azikiwe University, Awka, Nigeria; 4 Directorate of Research, Publications and Consultancy, University of Dodoma, Dodoma, United Republic of Tanzania; 5 Deputy Principal’s Office of Planning, Finance and Administration, The Law School of Tanzania (LST), Dar es Salaam, United Republic of Tanzania; 6 Health Promotion Unit, Ministry of Health, Dodoma, United Republic of Tanzania

**Keywords:** COVID-19 vaccination, social dilemma, social influence, hesitancy, acceptance, Tanzania

## Abstract

**Background:**

COVID-19 vaccination hesitancy and acceptance remain critical public health concerns, influenced by socio-cultural factors globally. Social influence – particularly conformity, compliance and obedience – influence vaccination intentions, decisions and behaviours based on the information circulated by the people already vaccinated. Understanding these dynamics is essential for promotion vaccine uptake through reassuring the hesitant about the safety and effectiveness of the vaccine.

**Aim:**

We explored the influence of social influence on COVID-19 vaccination hesitancy and acceptance in Tanzania.

**Setting:**

Our study was conducted in eight regions of Mainland Tanzania: Arusha, Morogoro, Mtwara, Njombe, Mbeya, Tabora, Singida and Shinyanga, to represent eight zones of Mainland Tanzania.

**Methods:**

We adopted a mixed-methods research approach, to collect data from 3098 respondents for a quantitative part, and 336 key informants as well as 376 participants for focus group discussions. Data analysis involved both descriptive and inferential statistics for quantitative data as well as thematic analysis for qualitative data.

**Results:**

There was regional variation in vaccination rates, with Mtwara and Singida showing high acceptance at 50% and 49.7%, respectively, while Morogoro (22.5%) and Mbeya (26.2%) showed lower rates. Social influence, especially friends, family, and community discussions, and trust in the government as a reliable source of information regarding the COVID-19 vaccination were key. Changes in the Tanzanian government’s political will also contributed to positive attitudes regarding COVID-19 vaccination acceptance.

**Conclusion:**

Social influence influenced COVID-19 vaccination acceptance in Tanzania, requiring tailored public health strategies involving the government, trusted community figures and considering social ties and social interaction to boost vaccination rates.

**Contribution:**

Our study offers insights on the critical role of social influence on COVID-19 vaccination hesitancy and acceptance; hence, a necessity for socio-cultural context-specific and participatory interventions in a quest to reduce COVID-19 vaccination hesitancy and improve acceptance in the Tanzanian context.

## Introduction

The World Health Organization (WHO) declared the COVID-19 outbreak a public health emergency in January 2020, leading to measures such as lockdowns, quarantines, social distancing and vaccinations.^1,2^ Moreover, the rapid development and approval of COVID-19 vaccines raised some debates over their safety, despite the fact that vaccination is crucial to lessening the impact that the virus had on communities.^3^ However, African countries, including Tanzania, expressed vaccination hesitancy because of doubts over the reliability of new vaccines, despite advancements in COVID-19 vaccines.^3,4,5^ Moreover, the COVID-19 vaccine hesitancy in Africa was fuelled by the COVID-19 vaccination campaigns against it that sparked intense discussions on social media, with prominent religious and political figures joining the debate. For instance, the former president of Tanzania, the late Dr John Pombe Magufuli who, from the earlier beginning of COVID-19 pandemic underestimated its severity and seriousness, strongly opposed the adoption of vaccination against this pandemic. Instead, he consistently emphasised the need for the Tanzanians to pray hard and use traditional medicines as the right strategy in the Tanzanian context.^6^ Later on, however, Tanzania managed to vaccinate 14% of the population since July 2021, and over 90% of individuals aged 18 years and above had received the vaccine by December 2022.^7,8^ As of April 2023, Tanzania was recognised as the top-performing country among 34 African nations in receiving coordinated assistance from the COVID-19 Vaccine Delivery Partnership.^5,9,10^

Given the observed patterns of COVID-19 vaccine adoption in Tanzania, it is critical to acknowledge that the decision to embrace the new vaccination is inherently intricate and open to many interpretations among the intended recipients.^11^ For example, the spread of conspiracy theories and false or misleading information regarding COVID-19, particularly the vaccination, might have caused people to hesitate, whereas the opposite may have led to acceptance,^12,13^ which brings to the fore the issue of social influence in respect of acceptance and hesitancy (a manner in which individuals adjust or change their opinion, their beliefs or their behaviour as a result of social interactions with other people).^14^ Thus, this study explored the influence of family members and friends, constituted authorities, community leaders and healthcare workers on COVID-19 vaccination in Tanzania.

## Research methods and design

### Study design

We employed a mixed-methods research approach, integrating both qualitative and quantitative data. Qualitative data were collected through focus group discussions (FGDs) and key informant interviews (KIIs), while quantitative data were gathered through household surveys to statistically assess the extent of hesitancy and acceptance across socio-demographic variables, vaccination status and information sources.

### Study setting

We conducted this study in eight regions of mainland Tanzania: Arusha, Singida, Shinyanga, Morogoro, Tabora, Njombe, Mbeya and Mtwara. Three districts – representing urban, semi-urban, and rural areas – were purposively selected within each region, totalling 24 districts. In each district, two wards (one urban, one rural) were randomly selected, allowing the study to encompass a diverse range of community settings across Tanzania.

### Study population and sampling

The study targeted community members aged 18 years and older who had lived in the selected communities for at least 18 months. A sample of 3098 respondents, 336 KIIs and 376 participants for FGDs from eight regions of Mainland Tanzania provided the data. A multi-stage convenience sampling procedure was adopted to collect data from a vast and geographically spread group of individuals. The Regional Health Management Teams assisted in recruiting local guides for house-to-house surveys. Participants were engaged through multiple avenues, including outreach at regular immunisation clinics, postnatal clinics and within the communities.

### Data collection

We collected the quantitative data using household micro-surveys, and qualitative data using FGDs and KIIs. While FGDs were conducted to understand attitudinal responses at group levels, KIIs generated in-depth discussions on the social factors that influenced COVID-19 vaccination intentions and actions. The KIIs included two religious leaders and/or tribal leaders depending on the district, one political leader who was either a councilor [*diwani*] or member of parliament [*mbunge*] or ward or village leader and a teacher and/or another influential person. For the purpose of establishing heterogeneity, we collected data on socio-demographic variables such as age, gender, marital status, religion, income level, education level and location of residence alongside data regarding patterns and the role of social influence on COVID-19 vaccination hesitancy and acceptance. Study participants were approached face-to-face through multiple avenues including outreach at regular immunisation clinics, postnatal clinics, and within the community.

### Data analysis

Qualitative data from FGDs and KIIs were transcribed, translated and coded using NVivo 14 Software (NVivo International Pty Ltd, Melbourne, Victoria, Australia). The analysis followed thematic analysis based on emerging themes. Quantitative data, analysed with Statistical Package for Social Sciences (SPSS 27.0 Software: IBM Corporation, Armonk, New York, United States [US]), underwent data cleaning and cross-tabulation to explore relationships between socio-demographic variables and vaccination attitudes. The results were integrated to present a comprehensive understanding of the factors influencing COVID-19 vaccination behaviours.

### Ethical considerations

Ethical approval to conduct this study was obtained from the University of Dodoma Institutional Research Review Ethics Committee (IRREC) (No. MA.84/261/76/214). We obtained the research clearance to conduct this study from the President’s Office-Regional Administration and Local Government (PO-RALG) in Mainland Tanzania and from the Ministry of Health (MoH). Permission was also sought and obtained from the Regional Health Management Teams (RHMTs) at the regional level and at each District/Council’s Health Management. Audio-recorded verbal consent was obtained from all the participants before undergoing any research procedures. Permission to record all the FGDs and KIIs was requested from all the participants before the interviews. Participants were assured that personal names would not be used anywhere in the analysis and reporting to ensure privacy and confidentiality of information. In addition, participation in this study was voluntary and participants were given the freedom to withdraw from the study at any point of their convenience.

## Results

### Participants’ socio-demographic characteristics

Our study involved a predominantly youthful sample, with the dominant age group being individuals aged 30–39 years (28.1%). This implies that younger people may be more likely to engage in discussions on COVID-19 vaccination, possibly because of social media and peer influence. Older age groups were under-represented, possibly because of reduced engagement or logistical challenges. The study also revealed a higher proportion of female respondents (56.7%), suggesting the need for gender-specific communication strategies in vaccination campaigns. The majority of the participants identified as Christians (59.3%), with Muslims representing 38.7% (see Table 1). This highlights the importance of religion in public health contexts, as religious beliefs and guidance can influence vaccination acceptance.

**TABLE 1:** The socio-demographic characteristics of the participants (*N* = 3098).

Characteristics	Frequency (*n*)	%
**Age (years)**		
18–29	689	22.2
30–39	871	28.1
40–49	645	20.8
50–59	438	14.2
> 60	455	14.7
**Gender**		
Female	1757	56.7
Male	1341	43.3
**Religions**		
Christian	1837	59.3
Muslim	1198	38.7
Other	63	2.0
**Educational level**		
No formal education	296	9.5
Primary education	1917	61.9
Secondary education	698	22.5
University/Tertiary	114	3.7
Vocational Education	73	2.4
**Marital status**		
Divorced/Separated	169	5.5
Married	2067	66.7
Other	5	0.2
Single	624	20.1
Widowed	233	7.5
**Income level per month (in TZS)**		
< 100 000	1728	55.8
100 000–250 000	1000	32.3
250 000–500 000	276	8.9
500 000–1 000 000	73	2.4
1 000 000 and above	21	0.7
**Location of residence**		
Rural	1342	43.3
Semi-urban	779	25.2
Urban	977	31.5

TZS, Tanzanian shillings.

The participants predominantly had primary education (61.9%), with secondary education holding 22.5%. Only 3.7% have tertiary or vocational education (2.4%). This implies that limited formal education might have hindered understanding of medical information, increasing vulnerability to misinformation. Clear public health messaging is therefore, essential for effectiveness and sustainability of interventions. Nevertheless, the majority of participants were self-employed (68.8%). The low employment levels in government and private sectors reflect the country’s economic structure, with self-employment being common. This suggests that limited financial resources might hinder vaccination uptake, as individuals may prioritise health decisions based on their financial status and priority. Therefore, addressing financial concerns within public health messages could reduce COVID-19 vaccine hesitancy and support increased vaccine acceptance.

### COVID-19 vaccine uptake status

**FIGURE 1:**
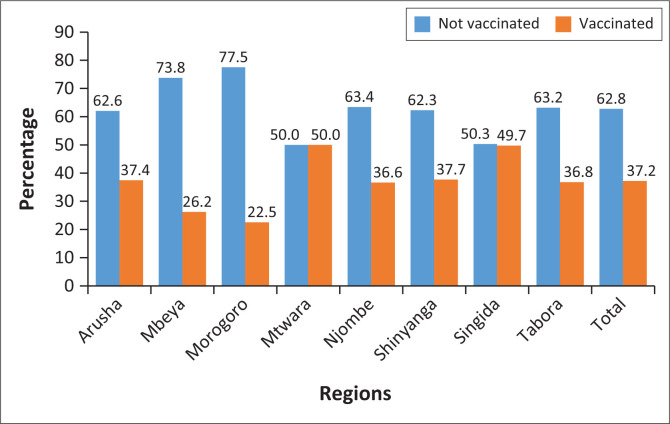
Coronavirus disease vaccination uptake status in selected eight regions of Tanzania.

As far as the COVID-19 vaccination was concerned, the highest and most immediate social benefit from the COVID-19 vaccination was the attainment of herd immunity. In this study, the status of the vaccination rate is as summarised in Figure 1.

Figure 1 shows significant regional variances in vaccination rates, with Mtwara and Singida having the highest rates of COVID-19 vaccination at 50.0% and 49.7%, respectively, indicating a rather balanced vaccination situation in these regions. On the other end of the spectrum, Morogoro has the lowest vaccination rate (22.5%), followed by Mbeya (26.2%). These findings imply that a significant section of the populace in these areas did not receive vaccinations. The vaccination rates in other areas, especially Arusha, Njombe, Shinyanga and Tabora, ranged from 36.6% to 37.7%; these were below the national average but not as low as those in Morogoro and Mbeya. It is crucial to remember that the percentages in the figure correspond to the entire population in each region who took part in the survey, not the total number of research participants.

### Participants’ socio-demographic characteristics and COVID-19 vaccination hesitancy and acceptance

As Table 2 indicates, we found a significant association between gender and vaccination status for COVID-19 (*p* < 0.05), suggesting that men and women differ in their propensity to receive the vaccine. Gender-specific perceptions of the vaccine, health priorities or cultural expectations could influence this. Gender-sensitive interventions could be effective, such as health campaigns targeting women to address vaccine safety concerns and men to emphasise the community role of vaccination. Education level also showed a significant association with vaccination status with a *p*-value of 0.0006, suggesting that individuals with lower levels of education may have limited access to reliable vaccine information or be more susceptible to misinformation. Addressing these disparities may require targeted educational campaigns and community-based outreach.

**TABLE 2:** Chi-squared and Kruskal-Wallis tests for gender, education, religion and age with respect to COVID-19 vaccination status in Tanzania.

Characteristic	Test statistic	*p*	*df*	Expected frequencies	Implication
Gender	11.45†	0.0033	2	Varies per category	Significant association with vaccination status
Education	19.55†	0.0006	4	Varies per category	Significant association with vaccination status
Religion	25.00†	< 0.0001	2	Varies per category	Significant association with vaccination status
Age	14.59‡	0.0001	N/A	N/A	Significant difference between age groups

N/A, not applicable; *df*, degrees of freedom.

† , Chi-square;

‡ , Kruskal-Wallis.

Religion had a strong correlation with vaccination behaviour, with a Chi-square value of 25.00 and a p-value of less than 0.0001, indicating its influence on health decisions. This implies that faith-based influences, particularly in collectivist settings, might shape individual behaviour. Partnerships with religious leaders and institutions could help increase vaccine acceptability. Health campaigns utilising religious figures and meetings to distribute accurate vaccine information could boost public trust and vaccination rates, particularly in regions where religious guidance is important (see Table 2).

Nevertheless, there were significant differences in vaccination status across age groups. Age differences in vaccination rates are influenced by factors such as perceptions of COVID-19 risk, vaccine accessibility and initial prioritisation during rollouts. Younger age groups may exhibit hesitancy because of lower risk perceptions, while older adults may be more accepting. Understanding these differences allows health officials to tailor messaging, promoting vaccine safety and community responsibility for younger people and protection from severe disease for older adults.

Therefore, the COVID-19 vaccination decisions among Tanzanians are influenced by factors such as gender, education, religion and age. These factors can influence vaccine uptake, highlighting the need for targeted public health strategies. Recognising gender differences in vaccine uptake can help identify unique barriers for men and women. Clear communication strategies, particularly in regions with lower education, can counter misinformation and improve vaccine understanding. Collaborating with religious institutions can enhance vaccine acceptance and reduce hesitancy. Tailoring messages based on age-related concerns could also help address diverse motivations and barriers across age groups.

### The role of social influence on COVID-19 vaccination hesitancy and acceptance

**FIGURE 2:**
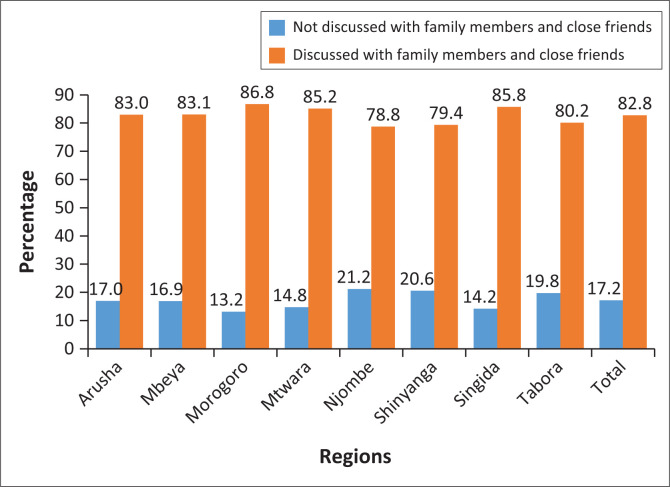
Discussion patterns with family members and close friends among participants regarding COVID-19 vaccination.

We found that talks concerning COVID-19 vaccination are common across eight regions where our study was confined, with an average of 82.8% of respondents participating. This shows that social impact could considerably shape people’s attitudes towards vaccination, as talks within intimate networks frequently reinforce beliefs and influence personal health decisions. Regional disparities in conversation rates reflect variations in social dynamics as well as the influence of local leaders or regional health programmes. Morogoro and Singida have the greatest conversation rates (86.8% and 85.8%, respectively), which could be attributed to local awareness initiatives, proactive roles by community or religious leaders, or increased worry about COVID-19. Njombe and Shinyanga had slightly lower discussion rates, which could be attributed to fewer public conversations, changes in public health communication techniques or different degrees of access to trustworthy information related to the COVID-19 vaccination (Figure 2).

The qualitative data align closely with the survey findings, reinforcing the critical role of social influence on COVID-19 vaccination acceptance among Tanzanians. We found that social influence shapes COVID-19 vaccination acceptance among Tanzanians. Family members, community leaders and government authorities shape attitudes towards vaccination, contributing to individuals’ decisions. Family networks play a crucial role in health decisions, as highlighted by participants in FGDs and KIIs. For instance, one participant from Kanyenye Ward in Tabora noted:

‘Many people influenced me, including those leaders who were vaccinated for the first time, and also the people who I was living with, my elders [*parents*] were also vaccinated, and I saw the need for getting vaccinated.’ (FGD, Community member 4, male, Tabora)

This reflects the role of social learning, where individuals model behaviours observed in trusted family members. Here, the vaccination status of parents and elders, who are often respected decision-makers within Tanzanian households, appeared to inspire confidence and normalise the decision to vaccinate among other family members. The influence of community leaders was also evident. In rural and semi-urban areas, local government leaders are often seen as both authoritative figures and role models. As one of the local government representative from Mbuguni Ward in Arusha stated:

‘I serve as both an ambassador and a village government representative in Mikungani. We have successfully encouraged numerous individuals to get vaccinated and plan to continue these efforts.’ (KII, Local government leader 7, male, Arusha)

This statement points to the active involvement of community leaders in vaccine advocacy, leveraging their positions to foster trust and promote public health within their communities.

Government influence is highlighted as particularly significant, with community leaders receiving attention and resources from authorities to support vaccination campaigns. A key informant from Mbuguni Ward in Arusha also noted:

‘The government ranks highest in terms of influence; they give considerable attention to us as community leaders at the village level.’ (KII, Community member 13, male, Arusha)

This statement underscores the government’s structured approach to utilising local government authorities and existing community structures to drive vaccine uptake, a strategy that aligns with survey findings linking higher vaccine acceptance to regions with active local governmental engagement.

Religious organisations also emerged as influential entities. Churches, mosques and even traditional healers hold respected positions within Tanzanian society, often guiding public opinion on health matters. As noted by one of the key informant from Tabora region, ‘Next in line are religious organisations like churches and mosques,’ (KII, Community member 14, female, Tabora) emphasising the value of religious institutions in lending credibility to healthcare-related campaigns. This finding suggests that, in areas where vaccine hesitancy is higher, involving religious leaders in promoting vaccination may help bridge trust gaps and counter misinformation effectively. Nevertheless, the healthcare workers were frequently cited as primary influencers in the community vaccination efforts. The village representative from Mikungani affirmed, ‘Healthcare workers are the main ones who visit us to sustain service provision’ (KII, Village representative 21, female, Arusha). This indicates that healthcare workers play a dual role of providing the vaccine and serving as credible, ongoing sources of information and reassurance. By consistently engaging with communities, healthcare workers appear to strengthen the acceptance and normalisation of vaccination.

### Trust in COVID-19 information from close social circles

As Table 3 reveals, Tanzanians across eight regions exhibited high trust in COVID-19 information communicated through their social circles, notably from friends, family and close contacts. The statistics indicate geographical variance, indicating that social context has a considerable influence on how information is received, processed, and interpreted. The Tabora and Shinyanga regions had higher levels of people trusting information from close social circles, with 42.3% and 40.7% of respondents believing this information totally. This trust may be linked to stronger, more coherent social networks in which people rely on one another for advice on health issues. Leveraging social networks could be a successful technique for public health campaigns in these areas.

**TABLE 3:** Level of trust about COVID-19 information from your friends, family members and colleagues, region-wise.

Region	Level of trust (%)				
	A little	Completely	Not at all	Not sure	Sometimes
Arusha	21.2	15.6	11.7	14.5	36.9
Mbeya	29.5	11.1	5.3	22.3	31.8
Morogoro	18.6	20.5	3.3	8.5	49.0
Mtwara	21.7	13.6	2.6	22.1	40.0
Njombe	37.6	12.3	4.3	14.6	31.2
Shinyanga	10.0	40.7	5.4	12.2	31.7
Singida	14.5	30.8	4.9	12.4	37.3
Tabora	6.1	42.3	4.5	10.3	36.8

**Total**	**20.2**	**23.0**	**5.2**	**14.9**	**36.7**

The data further show a moderate level of trust in information from social ties, with 23.0% of respondents completely trusting it and 36.7% trusting it ‘sometimes’. This suggests that social circles may not always be a reliable source of information. Morogoro has the highest percentage of respondents in the ‘sometimes’ category (49.0%), suggesting scepticism or conditional acceptance. Public health initiatives in Morogoro could benefit from integrating health experts or community leaders to provide clear, scientifically backed information. Some regions, such as Mbeya and Njombe, have lower levels of trust in socially shared information about COVID-19, possibly because of concerns about the credibility or accuracy of the information. These regions may be more susceptible to misinformation, as people may have encountered contradictory messages or witnessed adverse events that shaped their perceptions negatively.

In regions such as Mbeya and Njombe, health campaigns could prioritise dispelling misinformation and promoting information transparency. Empowering trusted community figures to provide fact-checked information backed by healthcare authorities can help mitigate lingering doubts. A significant portion of respondents expressed uncertainty or mistrust towards information shared through social ties, with 14.9% ‘not sure’, 20.2% ‘a little’, and 5.2% ‘at all’. This mistrust may stem from mixed messages within communities or previous experiences with health campaigns. Public health strategies could involve more targeted information sessions involving healthcare professionals to answer community questions and concerns directly.

### Social perception on the government as a reliable source of COVID-19 information (compliance and obedience to authority)

The Tanzanian government’s reliability as an information source on COVID-19 vaccination was generally perceived positively, with 86.2% of respondents viewing it as trustworthy. This high level of trust suggests that government-led initiatives and health communications have positively influenced public opinion. Regional variations in trust levels are evident, with Singida having the highest rate of strong agreement at 49.7%, possibly because of localised communication efforts or perceived responsiveness by government representatives. Morogoro had the highest level of agreement at 60.3%, possibly because of consistent and accessible government engagement with local communities. Mbeya has a lower rate of strong agreement at 20.9% and the highest neutral response rate at 21.1%, possibly because of less consistent government presence (Table 4).

**TABLE 4:** Perceived reliability of government as a source of COVID-19 information (compliance and obedience to Authority) (*N* = 3098).

Region	Level of agreement (%)				
	Agree	Disagree	Neither agree nor disagree	Strongly agree	Strongly disagree
Arusha	49.2	0.8	7.3	41.6	1.1
Mbeya	55.0	2.1	21.1	20.9	0.9
Morogoro	60.3	1.4	9.3	28.5	0.5
Mtwara	49.3	3.1	15.0	32.6	-
Njombe	45.8	3.8	14.8	33.5	2.0
Shinyanga	43.9	0.5	9.8	45.3	0.5
Singida	42.2	0.5	6.7	49.7	0.8
Tabora	49.7	-	5.8	44.2	0.3

**Total**	**49.5**	**1.6**	**11.5**	**36.7**	**0.8**

Disagreement levels remain minimal across all regions, with Njombe and Mtwara having the highest disagreement rates. Overall, Tanzanians generally have a positive or neutral stance on the government’s role as a reliable information provider on COVID-19. These findings suggest that individuals may feel undecided about the reliability of government-sourced COVID-19 information, as they may not have been consistently exposed to such information. Neutrality may also be because of competing information from other trusted sources. In regions with high neutral response rates, individuals may seek verification from multiple sources before trusting government information.^15,16^ However, the 86.2% positive response rate highlights the importance of government communication in shaping public opinion on health-related issues. High levels of trust in government sources can influence vaccination behaviours and compliance, especially in regions where consistent messaging is visible.

The qualitative data revealed a complex linkage between authority figures and community perceptions of COVID-19 vaccination in Tanzania. Government, religious and community leaders were key influences, with respondents referring to both local and central leaders as trusted sources. Leaders set examples to alleviate public doubts. For example, a participant from Mbuguni Ward in Meru District, Arusha Region, stated:

‘The government ranks highest in terms of influence; they give considerable attention to us as community leaders at the village level. Next in line are religious organisations like churches and mosques. Additionally, traditional leaders and heads of community groups, such as “Vikoba VICOBA”, also wield significant influence these days.’ (FGD, Community member 21, female, Arusha)

This participant quote emphasises the hierarchical structure of influence, with the government at the top, highlighting its role in initiating and disseminating health information. It suggests a multi-tiered approach, collaborating across religious, governmental and community levels, for maximising vaccine acceptance. Similarly, a village representative from Mbuguni Ward emphasised community leaders’ roles in supporting health workers in vaccination campaigns:

‘I serve as both an ambassador and a village government representative in Mikungani. We have successfully encouraged numerous individuals to get vaccinated … Health workers are the main ones who visit us to sustain service provision. A lot of our community leaders have also played a pivotal role in advocating for vaccination.’ (KII, Village representative 1, male, Arusha)

This statement highlights the collaboration between government representatives and healthcare workers to promote vaccine uptake, emphasising the importance of example-setting to build community trust and alleviate fears. The qualitative responses reveal that compliance with COVID-19 vaccination efforts is heavily influenced by individuals’ trust in government authorities and community leaders, who are perceived as credible sources of information. The Village Chairperson at Mwawaza Ward, Shinyanga, described how his own vaccination led the community by example:

‘I was persuaded by the health department here in Mwawaza … I felt that as a leader if I don’t get vaccinated it would seem strange … we leaders were at the forefront to get vaccinated to make people have no worries.’ (KII, Village chairperson 7, male, Shinyanga)

This example demonstrates how visible actions by authority figures can build public trust, especially when these figures are seen as guardians of public welfare. The chairperson’s proactive stance reflects an understanding that their health decision impacts their credibility, which in turn influences public confidence in vaccination.

In a FGD in Karatu Ward, a participant spoke of their motivation to protect not just themselves but also their family and community:

‘I got vaccinated because I was motivated … I had to get vaccinated … I motivated my family, my wife to get vaccinated … I wouldn’t want to infect others.’ (FGD, Community member 59, female, Arusha)

The statement highlights the sense of social responsibility among community members, influenced by personal interactions and trusted figures. Compliance with vaccinations leads to wider acceptance within family and social circles. However, some respondents still show hesitancy, often because of unmet information needs or traditional decision-making practices. Some respondents expressed reluctance because of the lack of specific health worker information:

‘If I would have been told that if get vaccinated the vaccine will stay in my body for how long, I would be ready to vaccinate.’ (FGD, Community member 37, male, Mbeya)

This response highlights a gap in detailed health information that, if addressed, could reduce reluctance. It suggests that for certain individuals, factual clarity about the vaccine’s effects or longevity within the body could enhance compliance. A participant in Lupalilo Makete mentioned the traditional practice of consulting elders before health decisions, stating:

‘Here when a person is sick, they must ask the elders first … only nowadays people look down on the elderly, but in the past … they must ask the elderly first before going to the hospital.’ (KII, Community member 16, female, Njombe)

The generational tension between traditional customs and modern health practices in Tanzania was evident, with some community members favouring traditional guidance over modern health recommendations. However, this does not mean a complete rejection of vaccination, but emphasises the importance of culturally respectful engagement. Trust and obedience to government-led COVID-19 vaccination efforts are strong, with authority figures and community leaders playing key roles. To address hesitancy, public health campaigns should provide detailed information on vaccine properties and integrate respected traditional figures into health messaging. Overall, these insights underscore the importance of a collaborative, culturally attuned approach to vaccination campaigns, where government trust is reinforced through community-based, transparent and inclusive strategies.^17,18^

A prominent theme in the findings is the influence of the late President John Magufuli’s fifth regime, which discouraged COVID-19 vaccines and modern preventive measures. This stance deeply affected public attitudes, fostering initial scepticism and reluctance towards vaccination. A participant from Urambo District in Tabora reflected this perspective:

‘I personally cannot get vaccinated unless the government first reverses its first position under the late President Magufuli … If our government’s stance has shifted, we don’t object.’ (FGD, Community member 8, female, Tabora)

This sentiment reveals the lasting impact of governmental stance, as public trust in vaccination was diminished by initial government scepticism. When the government’s position changed under President Samia Suluhu Hassan, there was a corresponding increase in vaccination uptake, driven by heightened importation of vaccines and advocacy campaigns. For instance, a respondent from Ngero Ward, Singida, observed:

‘Under the sixth regime, the government’s confidence in vaccines has led to a surge in vaccination rates among us, including myself.’ (KII, Community member 59, female, Singida)

The government’s proactive approach to vaccination in the sixth regime led to increased trust in the vaccine’s safety and necessity. Local and central government leaders, who publicly endorsed vaccination or participated in health campaigns, acted as role models, reducing public hesitancy and highlighting the influence hierarchy in their communities:

‘[*O*]ur president, the late Dr. Magufuli was misinformed or was not aware that prayers alone could not stop the virus. It was more important and easy to contain the virus using these scientific ways like vaccination and we are happy that the sixth regime under president Samia was at the forefront promoting the vaccines and they themselves got vaccinated first. It is good that even the religious leaders adopted the prayer approach but were also very supportive with President Samia’s vaccination initiative.’ (KII, Community member 18, male, Mtwara)

This hierarchy underscores the role of the government in leading health initiatives, with support from religious and community leaders, suggesting that a multi-tiered approach involving respected figures at various levels can enhance compliance. Village leaders, such as the chairperson from Mwawaza Ward in Shinyanga, further strengthened public trust by setting personal examples of compliance. They became advocates for vaccination, as exemplified by this statement:

‘As a leader, if I don’t get vaccinated, it would seem strange … we leaders were at the forefront to get vaccinated to make people have no worries.’ (FGD, Village chairperson 6, male, Shinyanga)

This approach highlights a common public health strategy of enlisting local influencers to alleviate concerns and build trust in COVID-19 vaccination.

### Compliance and trust in government information through influential social groups

The KIIs revealed Tanzanians’ perception and response to COVID-19 vaccination information, with religious bodies, politicians and community leaders playing a crucial role in shaping public attitudes and compliance with vaccination directives.

Religious institutions significantly influence members’ attitudes and behaviours towards health-related matters, including vaccination, according to a KII from Iwawa Ward in Makete DC, posing challenges in communicating consistent health messages across different faith groups. One participant noted:

‘Christians have their own ideologies and are not allowed to interfere with those of Muslims.’ (KII, Community member 71, female, Njombe)

This division highlights the potential for religious ideologies to act as both a facilitator and a barrier to vaccination acceptance, depending on alignment with public health messages.

The importance of these bodies is further reinforced by the observation from a key informant in Shinyanga DC:

‘Leaders who have a great influence are those who come from Christianity … spreading the message to the Isela Magazi community and mosques even before the COVID vaccination began.’ (KII, Community member 310, female, Shinyanga)

Religious leaders and politicians played significant roles in promoting COVID-19 vaccination, fostering compliance and trust within their communities. Religious leaders distribute leaflets and make announcements in places of worship, while politicians engaged with diverse community groups and advocate for national health programmes. However, their influence can be complex because of regional political dynamics and ideologies. Political figures aligning with public health goals can boost public trust in vaccination initiatives, but inconsistent health messaging across political platforms is needed to maintain public trust. Nevertheless, local government leaders play a crucial role in promoting health initiatives, especially in areas where governmental messaging may not reach. Their trusted status and understanding of local sentiments help reinforce government directives, facilitating greater compliance. The level of compliance and trust varied depending on key social groups and healthcare outreach, suggesting the need for stronger public health communication.

## Discussion

Provided that in the medical records, it is widely acknowledged that no vaccine has been as dearly anticipated as that to protect against COVID-19 pandemic,^19^ the COVID-19 vaccination has medical, psychological and social implications especially on curbing the impact related to mortality. As stated earlier that in this study, we examined the social dynamics influencing COVID-19 vaccination hesitancy and acceptance in Tanzania, focussing on demographic factors, social influences and regional disparities. Vaccination rates were disparate across different regions, with Morogoro and Mbeya showing low rates, indicating the need for region and context-specific tailored interventions. Mtwara and Singida showed higher rates, suggesting effective communication strategies or community engagement. Our findings suggest that misinformation from family and friends or the loss of trust in health authorities may contribute to hesitancy, highlighting the need for public health campaigns.^20^ This has been echoed by previous research that has demonstrated that socio-demographic traits, false beliefs and/or rumours regarding the effectiveness of vaccines, safety issues, cost and socio-cultural elements may affect people’s readiness to be vaccinated.^21,22^

Notably, discussions with family and friends significantly influence vaccination decisions. Majority (82.8%) of our respondents engaged in discussions about vaccination, highlighting the influence of interpersonal communication. Family, peers and community leaders are key sources of information and motivation to vaccinate.^21^ The study also highlights the gender disparity in respondents, with a significant representation of females. The majority of respondents were from the 30–39 years age group, with a religious affiliation of Christianity. Low vaccination rates in regions such as Morogoro and Mbeya suggest unique barriers to vaccine acceptance, possibly because of misinformation, the lack of access or socio-cultural factors.

Nevertheless, there was a moderate trust in COVID-19 information from family and friends as indicated by 23.0% of participants expressing complete trust on such information. This suggests scepticism because of conflicting messages about the vaccine, making COVID-19 vaccination a social dilemma.^22^ Trust is crucial in public health communication, and building credibility through community engagement and transparent information dissemination is essential. Community leaders and healthcare providers are pivotal figures in the vaccination discourse.^23^ Despite 77.9% receiving information from healthcare providers, a significant proportion remained uninformed, particularly in regions such as Morogoro. Government and religious leaders significantly influence vaccination decisions, and political narratives have a profound impact on public health.^23,24,25^ Community leaders and healthcare workers have been instrumental in promoting vaccination, dispelling myths and encouraging individuals to consider vaccination as a community responsibility.^26^

As is often the case for any study, our study has some limitations, such as participant bias based on the subjectivity of how they reacted to the news and studies related to the COVID-19 pandemic and their varied levels of socio-economic status. Another limitation of this study was the sample distribution, which involved eight regions representing the four zones of the Mainland Tanzania. Therefore, the responses from participants in this study might not accurately represent those from potential participants in similar or different regions. To overcome these limitations, we tried to be objective as much as possible in recording and reporting the results obtained from the field.

## Conclusion

Provided the fact that vaccination is often regarded by scholars and healthcare experts as one of the achievements of public health,^27^ our study has revealed that the decisions regarding COVID-19 vaccination in Tanzania were shaped by the opinions and actions (behaviours) of close people including friends, family, community leaders, and healthcare workers among social ties, particularly regarding the safety and necessity of being vaccinated. Hence, the vaccination status across the eight regions was influenced by social norms, trust in healthcare systems, access to information and the socio-demographic factors such as gender, education, religion and age, alongside social influence (family members, friends, religious leaders, community leaders, healthcare workers, and trust in the government). Moreover, as what studies like that of Salali et al.^28^ noted, friends and family members play an important role in influencing the decision to get vaccinated among the people. We, therefore, conclude that public health efforts including COVID-19 vaccination campaigns and programmes could move beyond a uniform approach to vaccination promotion and awareness campaigns, considering these socio-demographic factors and the social influence (conformity, obedience and compliance). Moreover, trust in the government as a reliable source of information regarding COVID-19 vaccination and healthcare providers’ dissemination of reliable information about the vaccine safety and related benefits promotes its acceptance among the people. Community engagement, using trusted individuals, and clear information are, therefore, of paramount importance for increasing vaccination uptake, bearing in mind that Tanzania was reported as one of the countries having lower COVID-19 vaccination rates globally.^9^ Community leaders should also participate in vaccination campaigns and policy interventions should tackle obstacles such as rumours, disinformation and misinformation.

## References

[1] Güner R, Hasanoğlu İ, Aktaş F. COVID-19: Prevention and control measures in community. Turk J Med Sci. 2020;50:571–577. 10.3906/sag-2004-146 32293835 PMC7195988

[2] Dadras O, Alinaghi SAS, Karimi A, et al. Effects of COVID-19 prevention procedures on other common infections: A systematic review. Eur J Med Res. 2021;26(1):67. 10.1186/s40001-021-00539-1 34217366 PMC8253677

[3] Brown P, Waite F, Larkin M, et al. “It seems impossible that it’s been made so quickly”: A qualitative investigation of concerns about the speed of COVID-19 vaccine development and how these may be overcome. Hum Vaccine Immunother. 2022;18:1–8. 10.1080/21645515.2021.2004808 PMC8928812 35172678

[4] Makoni M. Tanzania refuses COVID-19 vaccines. Lancet. 2021;397(10724):566. 10.1016/s0140-6736(21)00362-7 33581810 PMC7906632

[5] Mfinanga SG, Mnyambwa NP, Minja DT, et al. Tanzania’s position on the COVID-19 pandemic. Lancet. 2021;397(10284):1542–1543. 10.1016/s0140-6736(21)00678-4 PMC8046415 33864748

[6] Myumbo L. This is why Magufuli was right to be COVID-19 vaccine-Hesitant: Lessons for responses to future pandemics. J Philos Ethics. 2023;5(1):26–37. 10.22259/2642-8415.0501004

[7] Kaijage JM, Josephine L. Public perceptions of COVID-19 vaccine efficacy among urban dwellers in Tanzania – A case of Temeke Municipal, Research Report 2023/09. Dar es Salaam: REPOA; 2023.

[8] Chilongola JO, Rwegoshola KM, Balingumu OH, Semvua HS, Kwigizile ET. COVID-19 knowledge, attitudes, practices, and vaccination hesitancy in Moshi, Kilimanjaro Region, Northern Tanzania. Tanzania J Health Res. 2022;23(1):1–12.

[9] From below 10 to 51 percent – Tanzania increases COVID-19 vaccination coverage | WHO | Regional Office for Africa [homepage on the Internet]. WHO | Regional Office for Africa; 2023 [cited 2024 Nov 19]. Available from: https://afro.who.int/countries/united-republic-of-tanzania/news/below-10-51-percent-tanzania-increases-covid-19-vaccination-coverage

[10] Dodd RH, Pickles K, Nickel B, et al. Concerns and motivations about COVID-19 vaccination. Lancet Infect Dis. 2021;21(2):161–63. 10.1016/S1473-3099(20)30926-9 33338440 PMC7832277

[11] Konje ET, Basinda N, Kapesa A, et al. The coverage and acceptance spectrum of COVID-19 vaccines among healthcare professionals in Western Tanzania: What can we learn from this pandemic? Vaccines. 2022;10(9):1429. 10.3390/vaccines10091429 36146507 PMC9503367

[12] Anand U, Jakhmola S, Indari O, et al. Potential therapeutic targets and vaccine development for SARS-CoV-2/COVID-19 pandemic management: A review on the recent update. Front Immunol. 2021;12:658519. 10.3389/fimmu.2021.658519 34276652 PMC8278575

[13] Pandher R, Bilszta JLC. Novel COVID-19 vaccine hesitancy and acceptance, and associated factors, amongst medical students: A scoping review. Med Educ Online. 2023;28:2175620. 10.1080/10872981.2023.2175620 36788502 PMC9930839

[14] Moussaïd M, Kämmer JE, Analytis PP, Neth H. Social influence and the collective dynamics of opinion formation. PLoS One. 2013;8(11):e78433. 10.1371/journal.pone.0078433 24223805 PMC3818331

[15] Nossier SA. Vaccine hesitancy: The greatest threat to COVID-19 vaccination programs. J Egyptian Public Health Assoc 2021;96:18. 10.1186/s42506-021-00081-2 PMC8256776 34224031

[16] Hassan W, Kazmi SK, Tahir MJ, et al. Global acceptance and hesitancy of COVID-19 vaccination: A narrative review. Narra J. 2021;1(3):e57. 10.52225/narra.v1i3.57 38450215 PMC10914054

[17] Dhandapani JP, Subburayan D. Myths and facts about COVID-19: The pandemic. Pondicherry J Nurs. 2021;14:46–47. 10.5005/jp-journals-10084-13101

[18] Asch SE. Studies of independence and conformity: I. A minority of one against a unanimous majority. Psychol Monogr Gen Appl. 1956;70(9):1–70. 10.1037/h0093718

[19] Guner R, Hasanoglu I, Aktas F. COVID-19: Prevention and control measures in community. Turk J Med Sci. 2020;50(9):571–577. 32293835 10.3906/sag-2004-146 PMC7195988

[20] Asch S. Studies of independence and conformity: I.A minority of one against a unanimous majority. Psychol Monogr Gen Appl. 1956;70(9):1–70.

[21] Bingham K. The UK government’s vaccine taskforce: Strategy for protecting the UK and the world. Lancet. 2021;397(10268):68–70. 10.1016/s0140-6736(20)32175-9 33125932 PMC7833709

[22] Pei R, Cosme D, Andrews ME, Mattan BD, Falk E. Cultural influence on COVID-19 cognitions and growth speed: The role of cultural collectivism. PsyArXiv [Preprint]. 2020. 10.31234/osf.io/fet6z

[23] Larson HJ, Jarrett C, Eckersberger E, Smith DM, Paterson P. Understanding vaccine hesitancy around vaccines and vaccination from a global perspective: A systematic review of published literature, 2007–2012. Vaccine. 2014;32(19):2150–2159. 10.1016/j.vaccine.2014.01.081 24598724

[24] Al-mohaithef M & Padhi BK. Determinants of COVID19 vaccine acceptance in Saudi Arabia: A web-based national survey. J Multidiscip Healthc. 2020;13:1657–1663. 10.2147/JMDH.S276771 33262600 PMC7686470

[25] Badur S, Ota M, Öztürk S, Adegbola R, Dutta A. Vaccine confidence: The keys to restoring trust. Hum Vaccines Immunotherap. 2020;16(5):1007–1017. 10.1080/21645515.2020.1740559 PMC7227637 32298198

[26] World Health Organization (WHO). Ten threats to global health in 2019 [homepage on the Internet]. Geneva: World Health Organization; 2019 [cited 2024 Oct 12]. Available from: https://www.who.int/news-room/spotlight/ten-threats-to-global-health-in-2019

[27] Dubé E, Laberge C, Guay M, Bramadat P, Roy R, Bettinger JA. Vaccine hesitancy: an overview. Hum Vaccines Immunotherap. 2013;9(8):1763–1773. 10.4161/hv.24657 PMC3906279 23584253

[28] Salali GD, Uysal MS, Bozyel G, Akpinar E, Aksu A. Does social influence affect COVID-19 vaccination intention among the unvaccinated? Evol Hum Sci. 2022;11(4):e32. 10.1017/ehs.2022.29 PMC10426110 37588925

